# The Impact of Whole Brain Global Functional Connectivity Density Following MECT in Major Depression: A Follow-Up Study

**DOI:** 10.3389/fpsyt.2019.00007

**Published:** 2019-03-01

**Authors:** Xiao Li, Huaqing Meng, Yixiao Fu, Lian Du, Haitang Qiu, Tian Qiu, Qibin Chen, Zhiwei Zhang, Qinghua Luo

**Affiliations:** ^1^Department of Psychiatry, The First Affiliated Hospital of Chongqing Medical University, Chongqing, China; ^2^Department of Anesthesiology, The First Affiliated Hospital of Chongqing Medical University, Chongqing, China; ^3^Department of Radiology, The First Affiliated Hospital of Chongqing Medical University, Chongqing, China

**Keywords:** depression, electroconvulsive therapy, brain, functional connectivity, fMRI methods

## Abstract

To explore the alteration of global functional connectivity density (gFCD) in depressive patients after modified electroconvulsive therapy (MECT) and analyze the relationship between gFCD and clinical outcome. Thirty-seven subjects were evaluated based on the diagnostic criteria of the International Classification of Diseases-10 (ICD-10), consisting of a depressive group (24 patients after follow-ups) and a healthy control group with 13 normal individuals. All participants received Hamilton Depression Scale (HAMD) scores and resting-state functional magnetic resonance imaging scans. The gFCD significantly increased in the posterior-middle insula, the supra-marginal gyrus and the dorsal medial prefrontal cortex (dmPFC) before MECT treatment compared to healthy controlled patients. The gFCD statistically expanded in the perigenual anterior cingulate cortex (pgACC), the orbitofrontal cortex bilaterally and the left-supra-marginal gyrus after MECT, and it decreased notably in the posterior insula. The gFCD in the pgACC and the right orbital frontal cortex of depressive group before MECT showed a positive correlation with HAMD scores with treatment. Conforming to the impact of gFCD in depressive patients after MECT, the aforementioned brain region may become an indicator of MECT effect.

## Introduction

Major depressive disorder (MDD) is one of the most common mental disorders; however, limited therapeutic options are available, creating an enormous individual and societal burden. An estimated 30% of patients with MDD still suffer from functional impairment and antidepressant drugs are only partially effective (1). Modified Electroconvulsive therapy (MECT) is known as a useful treatment for MDD and works by eliciting controlled, transient seizures in both acute and maintenance sessions (2). Several meta-analyses have confirmed the antidepressant effectiveness of MECT for depression (3–5).

However, not all patients respond to MECT. Approximately only 50% of patients experience remission when receiving right-unilateral MECT with optimal parameters, and the specific neural mechanism of action still remains unclear. Until now, the modulatory effect of MECT on brain functional connectivity density (FCD) has only been reported in a few studies. Some found the dorsal-lateral prefrontal cortex (DLPFC) was crucial for achieving a therapeutic response by MECT (6–8). Previous studies have shown symptom recovery in some regions, such as the amygdala or the subgenual anterior cingulated cortex (sgACC) (9, 10). Cano et al. (11) found that substantial intra-limbic functional connectivity (FC) decreases predicted a later increase in limbic–prefrontal FC, which could predict clinical improvement at the end of a course of ECT.

As a voxel-wise, data-driven method, functional connectivity density mapping (FCDM) is widely used to test the density distribution of whole-brain resting-state FC (12–14), such as resting-state global functional connectivity density (rs-gFCD). Rs-gFCD is been referred to as the level of centrality (15) or intrinsic connectivity contrast (16). For some neuropsychiatric disorders, rs-gFCD was suggested to be a biomarker (15, 17, 18). Increased FCDs shows the elevated number and strength of FC may indicate its important role for understanding the mechanism in these brain areas. Kandilarova et al. (19) found, according to spectral dynamic causal modeling, that significantly reduced strength of the connection from the MFG (i.e., dorsolateral prefrontal cortex) to the anterior insula was shown in patients, and a strong connection was found between the anterior insula and the amygdala. This research may be used to predict treatment response.

Neuroimaging research of ECT has particularly assessed brain function before and after treatment (20–22), but have not tried to characterize functional changes occurring at various treatment phases. These measurements may have important clinical utility as an outcome predictor (23) and may help to reveal the mechanism of antidepressant treatments.

In this study, we hypothesized that a complex interaction between MECT-induced gFCD changes and clinical improvement will emerge in patients with MDD. Therefore, to demonstrate this relationship, we assessed a group of patients with MDD and compared them to healthy controls. We used functional magnetic resonance imaging to examine developments in global functional connectivity density and assessed alterations in Hamilton depression scale (HAMD) scores during MECT. We measured gFCD in the depressive group before MECT and in the healthy controls, and after 8 courses of MECT, we tested gFCD in the depressive group again. The specific objectives of the study were as follows: [1) to assess changes in specific regions throughout the course of ECT and [2) to expose the relationship between ECT-induced gFCD changes and clinical response.

## Materials and Methods

### Participants

Twenty-four patients and 13 demographically similar healthy control subjects received informed consent forms for participation in the research, which was approved by the First Affiliated Hospital of Chongqing Medical University. All the methods followed relevant regulations. Diagnostic assessment and response were assessed by experienced psychiatrists in depression and relevant scales. All the patients would take a clinical assessment before ECT including routine blood testing, chest X-rays, and brain CT scans (11). They were all experiencing MDD as defined by the ICD-10 and were screened using the Hamilton Depression Scale (pre-ECT 31.3 ± 8.6; post-ECT8.58 ± 5.62; healthy-control 2.21 ± 1.25). Subjects were excluded if they: (i) had a neurological or serious physical condition or any history of alcohol or drug abuse, or any other somatic diseases, or morphological anomalies of the brain, (ii) had any surgically-placed electronic or metal materials that might interfere with fMRI assessment, (iii) slept while scanning and/or (iv) had head motion exceeding 3 mm in translation or 3 degrees in rotation. The Local Medical Ethics Committee of the First Affiliated Hospital of Chongqing Medical University reviewed and confirmed the study protocol. Written informed consent was obtained from all subjects.

### Electroconvulsive Therapy

The patients underwent modified bi-frontotemporal ECT which was conducted using a Thymatron (TM) DGx (Somatics LLC, Lake Bluff, IL, USA), which is the brief-pulse, constant-current apparatus at the psychiatry department of the First Affiliated Hospital of Chongqing Medical University (24). The first three courses of ECT took place on continued days and the left courses of ECT were performed every 2 days, and it would have a break on weekends. After eight courses, ECT was continued if depressive symptoms had not changed sufficiently, as determined by a clinician, with a maximum of 12 courses of ECT. The initial dosage was confirmed based on age, weight and sex. Anesthesia was induced with succinylcholine (0.5–1 mg/kg) and diprivan (1.5–2 mg/kg).

### MRI Data Acquisition

A 3.0 Tesla MRI system (GE Signa) was used to obtain imaging data in the First Affiliated Hospital of Chongqing Medical University. Patients were asked to close their eyes peacefully and to keep their heads stable throughout MRI process and keep awake. After the MRI scan, they would be asked whether they had fallen asleep during the process (24). Resting-state fMRI images were collected using the following EPI sequence: repetition time: 2000 ms; echo time: 30 ms; flip angle: 90°; field of view: 240 × 240 mm^2^; matrix: 64 × 64; slice thickness: 5 mm; and number of slices: 33 axial slices. Two hundred volumes were obtained, resulting in a 400s scan time, then 3D T1-weighted anatomical images were collected (repetition time: 8.35 ms; flip angle: 12°; echo time: 3.27 ms; field of view: 240 × 240 mm^2^; matrix: 256 × 256; slice thickness: 1 mm; and the number of sagittal slices is 156) (25).

### Pre-processing and Quality Control

Data Processing & Analysis of Brain Imaging (DPABI) was used to assess resting-state data (26). The first 10 volumes of the functional images were abandoned to account for signal equilibrium (27). Slice timing and head motion correction were conducted in sequence for the remaining time points. The covariates, including head motion, white matter signal and cerebrospinal fluid signal, were regressed out from the time series of every voxel. Here, the Friston 24-parameter model was used to regress out head motion effects. To decrease effects of high-frequency noise and low-frequency drift, a 0.01–0.1 Hz band-pass filter was used. The registered images were spatially normalized to the Montreal Neurological Institute (MNI) template. The images were resampled to 3-mm cubic voxels. To smooth the normalized images, a 6-mm full width at half maximum Gaussian kernel was used. Additionally, the scrubbing procedure was employed, excluding any volume with a frame-wise dependent value exceeding 0.5, with the two subsequent volumes and one preceding volume. Finally, normalization quality was monitored by checking the normalization images subject by subject (28).

### Functional Connectivity Density

We used the DPARSF toolbox to calculate the functional connectivity density (FCD) of each voxel. High FCDs indicates increased strength and number of the respective FC, showing its significance in the brain. Between all the brain voxels, Pearson's correlation coefficients were calculated, so that the whole-brain functional connectivity matrix for each subject could be constructed. The degree centrality maps were computed using 0.3 (we also used 0.4 as the threshold for determining edges and found the results was similar) as the threshold for determining edges (12). Thus, the whole-brain maps were obtained by computing the number of voxels where the connections with other voxels in the BOLD time series exceeded the threshold in a whole-brain weighted graph.

### Statistical Analysis

First, the two-sample *T*-Test was selected to test the group differences in gFCD between pre-ECT MDD patients and the control group in a voxel-wise manner by using a general linear model with age, gender, and the motion (Mean FD) as nuisance covariates. A correction for multiple comparisons was performed using *p* < 0.05 with family-wise error (FWE), which is correct at the voxel level. Second, to investigate the therapeutic effect of ECT, the paired sample *T*-test was also used to test the differences between MDD before treatment and after treatment using ECT in a voxel-wise manner. Because the results may be easily impacted by noise, a conservative statistical threshold was specified at cluster level *p* < 0.05, which is correct with an underlying voxel level of *p* < 0.001(AlphaSim corrected) using DPABI (26) software. Additionally, one-way ANOVA was performed to test the difference in the ROI regions based on previous paired sample t results (pre-treatment vs. post-treatment) among the healthy controls, pre-treatment MDD and post-treatment MDD.

The statistical analysis of one-way ANOVA was implemented by SPSS 20 (IBM SPSS Statistics for Windows, Version 20.0, IBM Corp, Armonk, NY, USA). To characterize the relationship between HAMD scores and pre-/post- treatment, we computed Pearson's correlation analysis. Each group was compared with others by using a Bonferroni *post hoc* test with *p* < 0.05.

## Results

### Demographic Data and Psychological Measurements

The psychological measurements and demographic data are listed in Table 1. In comparison with the healthy controls and post-treatment depressive subjects, the pre-treatment depressive subjects had more serious depressive symptoms according to HAMD scores. One-way ANOVA analyses indicated that the scores on the HAMD were remarkably different among the three groups (*F* = 232.4, *p* < 0.001). A pairwise comparison found that post-treatment periods were characterized by significantly lower depressive symptoms than pre-treatment intervals (*F* = −22.8, *p* < 0.001), yet subjects in the post-treatment stage still experienced markedly higher depressive symptoms compared to healthy control subjects (*F* = 6.4, *p* < 0.001). The depressive group and the healthy controls did not differ considerably with age (*t* = −0.55, p = 0.59) or sex (*t* = −0.81, *p* = 0.43). Compared with the healthy controls, the depressive patients had more years of education (*t* = −3.7, *p* < 0.001). The individual scores from the HAMD of all participants are shown along with the mean in Table 1, excluding 6 patients who refused further treatment because of symptom recovery at an early stage.

**Table 1:** The demographic data and psychological measurements of the healthy controls, pre-treatment and post-treatment depressive subjects.

**Characteristic**	**Pre-treatment**	**Post-treatment**	**Control**	**Value**	***P***
Age, mean (SD), y	32.5 (11.7)	/	33.3 (10.4)	*t* = −0.55	*p* = 0.59
Sex (male/female)	10/14	10/14	5/9	*t* = −0.79	*p* = 0.43
^*^Education years, mean (SD), y	11.1 (2.86)	/	15.1 (3.47)	*t* = −3.9	*p* < 0.001
Body Weight, mean (SD), Kg	54.1	N/A	57.2	*t* = −1.46	*p* = 0.15
HAMD, mean (SD)	31.3 (8.6)	8.58 (5.62)	2.21 (1.25)	*F* = 164	*p* < 0.001
Head Motion (FD), mean (SD)	0.089 (0.03)	0.113 (0.09)	0.104 (0.03)	*F* = 1.54	*p* = 0.23

* *Results are P < 0.05*.

### Significant Differences Between Pre-treatment Periods and Healthy Controls in Global Functional Connectivity Density (gFCD)

**Figure 1:**
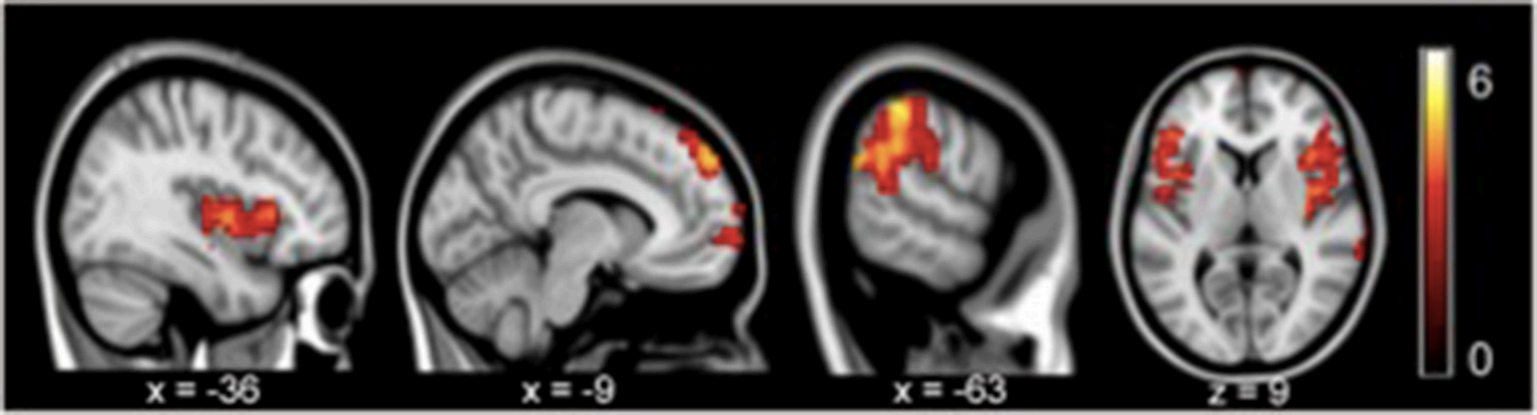
gFCD in pre-treatment revealed significant increases in the posterior-middle insula, supramarginal gyrus, and dorsal medial prefrontal cortex compared to the controls (*p* < 0.05, FEW).

In contrast with the healthy controls, the depressive patients in the pre-treatment phase exhibited a significantly increased gFCD in the posterior-middle insula, supra-marginal gyrus, and dorsal medial prefrontal cortex (all *p* < 0.05 with family wise error corrected, Figure 1). No regions showed decreased gFCD under the same statistical threshold.

### Significant Differences Between Pre-treatment and Post-treatment Stages in Global Functional Connectivity Density (gFCD)

**Figure 2:**
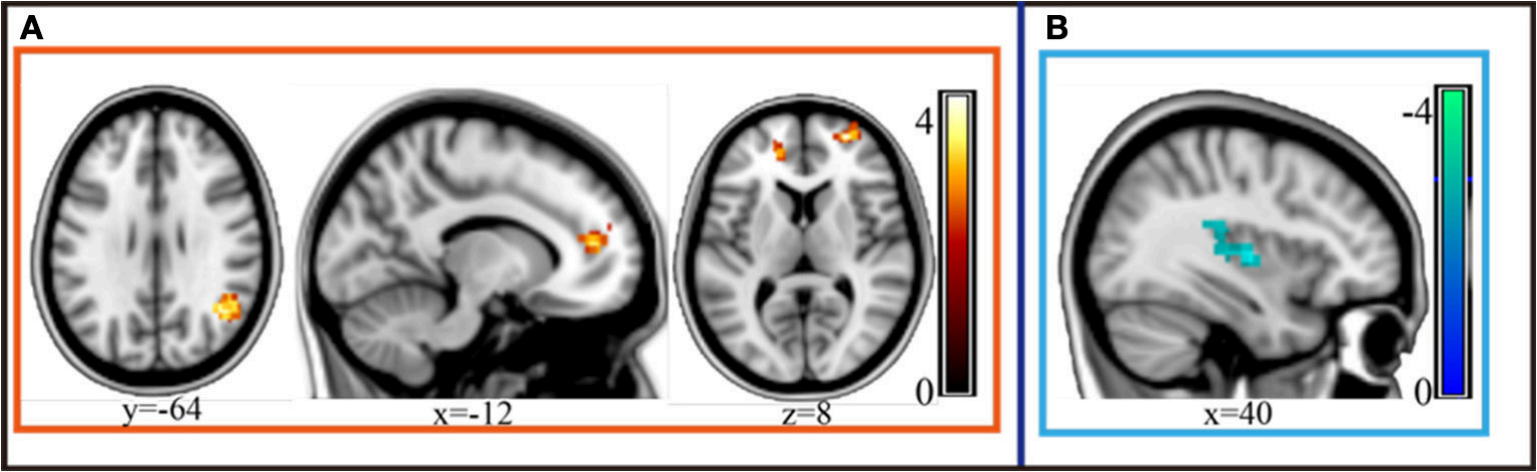
gFCD of the depressive group statistically increased in the perigenual anterior cingulate cortex (pgACC), orbitofrontal cortex bilaterally and the left-supramarginal gyrus after MECT (*p* < 0.05, FEW). **(A)** Statistical increased gFCD in these regions post treatment. **(B)** Statistical decreased gFCD in these regions post treatment.

Compared to the depressive patients in the pre-treatment period, the post-treatment depressive patients exhibited an obviously increased gFCD in the perigenual anterior cingulate cortex (pgACC), orbitofrontal cortex bilaterally and the left-supra-marginal gyrus. Moreover, depressive patients in the post-treatment stage exhibited decreased gFCD in the posterior insula compared to the pre-treatment phase (AlphaSim corrected *p* < 0.001 under voxel level uncorrected and cluster level under *p* < 0.05 familywise error corrected, Figure 2; Table 2).

**Table 2:** Significant difference between different groups in global functional connectivity density (gFCD).

**Brain regions**	**MNI coordinates**			**Voxel size**	**Peak T value**
	***x***	***y***	***z***		
**PRETREATMENT vs. CONTROL**					
**Increased gFCD**					
Middle insula (left)	−36	6	3	234	4.93
Posterior middle insula (right)	39	−3	3	310	4.87
Supramarginal gyrus (left)	−63	−51	30	296	5.68
Dorsal medial prefrontal cortex(DMPFC) (left)	−9	54	42	154	5.42
**Decreased gFCD**					
No					
**POST-TREATMENT vs. PRETREATMENT**					
**Increased gFCD**					
Perigenual anterior cingulate cortex (pgACC) (left)	−12	45	12	231	4.16
Right supramarginal gyrus (right)	54	−63	33	470	4.78
Orbitofrontal cortex (left)	−24	57	3	217	3.97
**Decreased gFCD**					
^a^Right insula (right)	36	−15	6	110	−4.03

a *Results are P < 0.05, corrected for multiple comparisons at a cluster level with AlphaSim, with an underlying voxel level of P < 0.001, uncorrected under whole brain analyses*.

**Figure 3:**
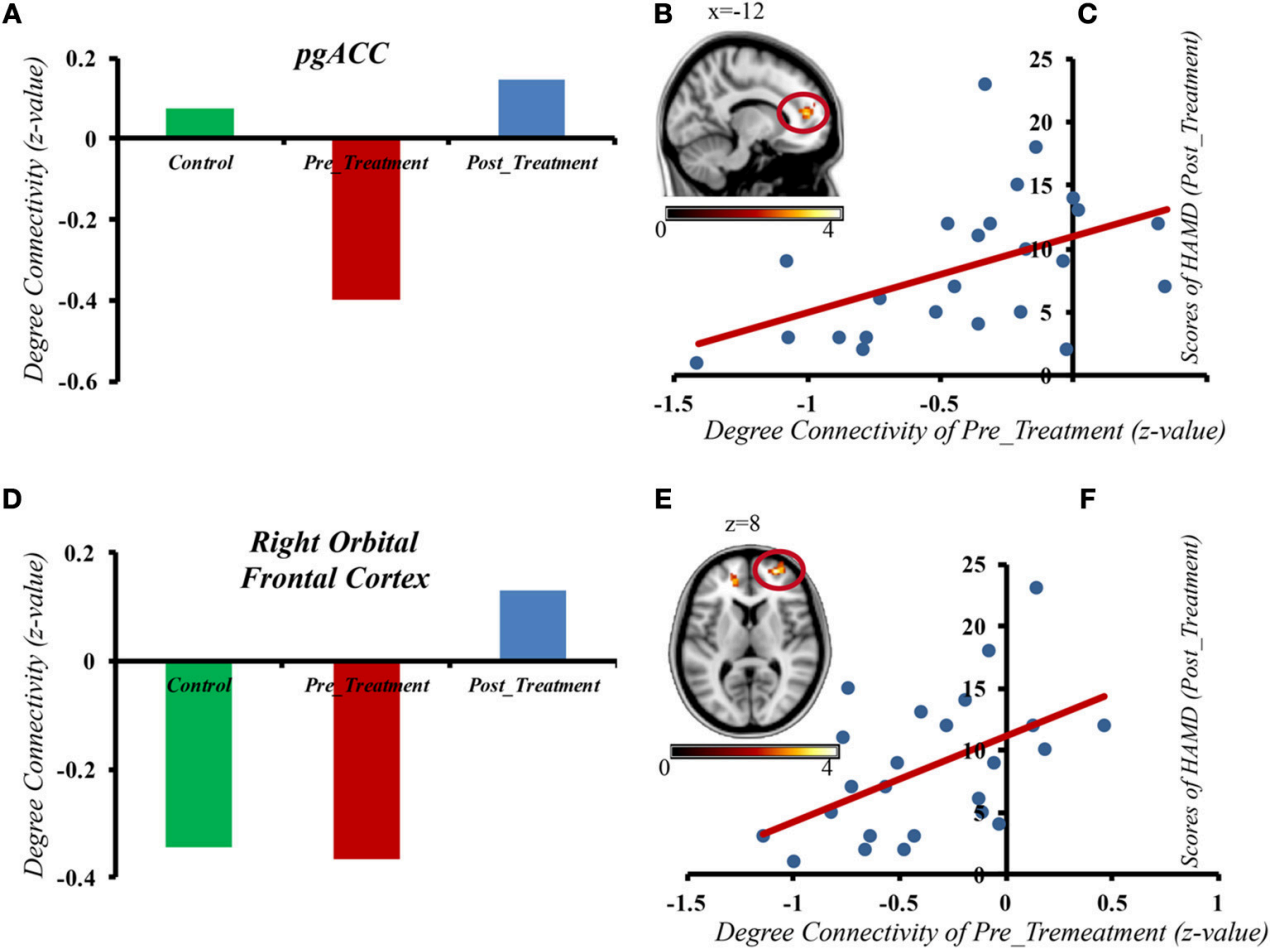
The gFCD in the pgACC and right orbital frontal cortex of depressive patients before MECT shows a positive correlation with HAMD scores after treatment. **(A)** gFCD in pgACC in three different groups. **(B)** gFCD in pgACC. **(C)** The gFCD in the pgACC of depressive patients pre MECT shows a positive correlation with HAMD scores post treatment. **(D)** gFCD in Right Orbital Frontal Cortex in three different groups. **(E)** gFCD in Right Orbital Frontal Cortex. **(F)** The gFCD in the Right Orbital Frontal Cortex of depressive patients pre MECT shows a positive correlation with HAMD scores post treatment.

The results from the correlation analyses revealed that the gFCD activity in the pgACC (*r* = 0.46, *p* = 0.024) and right orbital frontal cortex (*r* = 0.5, *p* = 0.013) in the pre-treatment interval had a significant correlation with the post-treatment period HAMD scores (see Figure 3). However, there was no correlation between the gFCD pre-MECT and HAMD scores pre-MECT (*p* > 0.05). There was also no noticeable correlation between post-treatment gFCD and HAMD scores. The changes of gFCD and the differences of HAMD scores also had no significant correlation. These results indicated that the gFCD activity present in pre-treatment may predict the post-treatment outcome.

## Discussion

In the present study, we assessed the gFCD changes in patients with MDD before and after MECT. The results demonstrated that, compared to the healthy controls, there was increased gFCD in the posterior-middle insula, supra-marginal gyrus, and dorsal medial prefrontal cortex of pre-ECT MDD patients.

The post-treatment results exhibited significantly increased gFCD in the perigenual anterior cingulate cortex (pgACC), orbitofrontal cortex bilaterally and the left-supra-marginal gyrus. These upshots were associated with decreased HAMD scores, and statistical analysis demonstrated that such connectivity changes were related to clinical outcome.

### Orbitofrontal Cortex

The orbitofrontal cortex (OFC) is important in complex human behaviors. OFC cortico-striatal circuits are consistently involved in many mental disorders, such as depression. Structurally, the OFC reveals remarkable decreased volumes in medication-naïve MDD patients compared to MDD patients who take medications (29). Additionally, Webb et al. (30) found higher depressive symptoms were related to reduce gray matter volume in the left rACC (extending into the OFC). Likewise, significant decreases have been observed not only in OFC gray matter, but also in the ventral striatum and amygdala in MDD patients (30, 31). Furthermore, the changes may last through the whole life. According to a previous study, no significant differences were observed in total gray matter volume of OFC, or in total OFC volume between MDD children and healthy controls (32). Interestingly, Rajkowska et al. (33) obtained post-mortem samples from elderly depressed patients that showed that the density of pyramidal neurons in the OFC was particularly low, which shows more severe neuronal pathology changes in older MDD patients than in younger patients. Studies show that, compared with control subjects, significant hyperactivity is observed in the mOFC and VMPFC for MDD patients (34–40). At the same time, some research shows that, for medication-naïve MDD patients, the resting cerebral blood flow (rCBF) of the OFC was upregulated, while after taking antidepressants, reduced metabolism was observed in these regions (41). Thus, there is a positive correlation with MDD patients. Nevertheless, the correlation between symptom severity and rCBF of these areas remains somewhat of a mystery. This research suggests that activation of these areas can be a complemental reaction for decreasing negative emotional action. Specifically, some results revealed an inverse correlation between decreased functional connectivity within the medial division of the orbitofrontal circuit and the severity of symptoms, which matches our conclusions (42).

### Insula

Some large trials demonstrated that pre-treatment regional insula activity could prognosticate the specific treatment that would be efficacious at the individual patient level. Dunlop et al. (43) found preliminary evidence that a putative right anterior insula metabolism biomarker could predict treatment outcomes, even in children (43). Belden et al. (44) found there was some kind of correlation between structural abnormalities of anterior insula volume and the neurobiology of depression from early childhood. Consequently, the function and structure of insula are significant for presaging the clinical outcomes of depression. Some previous studies indicated that low functional connectivity density in the insula leads to better clinical outcomes in MDD, but there is no research to reveal upregulated connectivity in ACC/VMPFC, PCC/pC, dACC and insula within RSNs that are correlated with MDD pathology. Regression results showed that areas related to clinical response overlapped mostly with areas that exhibited abnormal connectivity. ACC/VMPFC, dACC and the left insula are the hub areas of the default mode network (DMN) and SN. These areas displayed prominent performance (highest sensitivity = 100% and highest specificity = 82%) in distinguishing therapeutic effect (45). Some recent researches specified that, in contrast with MDD patients who not attempted suicide, those who have attempted suicide showed hyper-activity resting-state functional connectivity (RSFC) of the left amygdala with the right insula (46). Our corollary showed increased gFCD in the insula before treatment, which matches previous studies.

### Dorsal Medial Prefrontal Cortex

Chaotic network connectivity is observed in MDD core networks, which include DMN, of which the dorsal medial prefrontal cortex (dmPFC) is one part. Both pharmaceutical treatments and electroconvulsive therapy and repetitive transcranial magnetic stimulation can work in DMN. One previous study showed that, after using rTMS just in the dmPFC region, the symptoms became better (47). Previous studies have demonstrated abnormal changes in resting-state functional connectivity strength in several brain regions and brain networks (48–50); research has further shown that resting-state functional connectivity density is mainly located in the medial prefrontal cortex, posterior cingulated cortex, precuneus and occipital lobe. MDD patients show lower medial prefrontal cortex volumes. Research on a non-clinical sample found that, in the dorsal medial prefrontal cortex, male subjects with higher levels of depressive qualities seem to have lower volumes of gray matter (51). Even in a subclinical sample, the dorsal medial prefrontal cortex was shown to be a potentially significant biomarker for treatment outcomes in depression. In our study, we found increased gFCD in dmPFC in depressive patients. These outcomes match some previous research, such as how—compared with healthy control group—increased within-network connectivity was observed in the dmPFC of MDD patients (52), and another study showed increased resting-state FC between the medial prefrontal cortex and other DMN structures in patients who suffered from major depressive episodes (53). So, we predict increased gFCD exists not only in dmPFC its own, but also dmPFC with other brain regions.

### Perigenual Anterior Cingulate Cortex (pgACC)

Previous studies indicated that abnormal structure of the anterior cingulate cortex (ACC) is also frequently linked with major depression disorder (54–56). Dysfunction in networks including the ACC and caudate nucleus has been demonstrated to underlie many core symptoms of MDD such as anhedonia, decreased energy and intellectual disability (7). In addition, Wu et al. (57) found a remarkble reduction of functional connectivity strength (FCS) in sgACC in MDD patients. Taken together, this research supports a neurotrophic model of MDD and antidepressant effects, showing that ECT may cause functional alterations within prefrontal and limbic areas.

Furthermore, we also found that gFCD in the pgACC and the right orbital frontal cortex of depressive patients before ECT had a positive correlation with HAMD scores after treatment. Previous research has shown that the alteration of limbic and prefrontal networks is continuous during symptom remission. Early antidepressant effects can be observed at the limbic level, and the following effects can be observed in the PFC (6, 11, 58). Cano et al. (11) found that early, substantial decreased intralimbic FC significantly exhibited a subsequent increase in limbic–prefrontal FC, which meant better clinical outcomes could come from an ECT session. The gFCD in the pgACC and the right orbital frontal cortex of depressive patients before MECT showed a positive correlation with HAMD scores after treatment, which suggests that functional disturbances in MDD may be associated with compensatory activity enhancement in some regions. In severe depression, the compensatory enhancement is more obvious. Our results may provide evidence for finding a new predictor of treatment outcome.

In the present study, our research focuses on describing treatment outcomes with gFCD and MECT. However, previous studies have shown that regardless of function or structure, the regional insula, ACC, OFC, supra-marginal gyrus, and dorsal medial prefrontal cortex may be important biomarkers for treatment outcomes of depression. Some studies have shown decreased gFCD in the left occipital lobe of depressive patients while our study found that the gFCD in the pgACC and right orbital frontal cortex of depressive patients before MECT demonstrated a positive correlation with HAMD scores after treatment, suggesting that the level of gFCD in the pgACC and right orbital frontal cortex may also be core indicators of treatment outcome.

This study has some limitations. These include the small number of patients; replication with a larger sample is warranted and the healthy control sample was also small. We did not add a subgroup analysis and there were no other psychopathological or neurocognitive assessments in our research, and we did not follow the healthy controls; therefore, further research is needed.

## Conclusion

We found abnormal gFCD in the posterior-middle insula, supra-marginal gyrus, and dorsal medial prefrontal cortex in depressive patients after MECT. MECT influenced brain gFCD in depressive patients by increasing gFCD in the perigenual anterior cingulate cortex (pgACC), orbitofrontal cortex bilaterally and the left-supra-marginal gyrus while decreasing gFCD in the posterior insula after 8 courses of MECT. The gFCD in the pgACC and right orbital frontal cortex of depressive patients before MECT revealed a positive correlation with treatment outcome, demonstrating that the above brain region may be a strong indicator of MECT effect.

## Author Contributions

XL contributed to data collection and wrote the paper. HM, YF, LD, HQ, TQ, and QC helped revise the paper. ZZ and QL designed the experiment and revised the paper.

### Conflict of Interest Statement

The authors declare that the research was conducted in the absence of any commercial or financial relationships that could be construed as a potential conflict of interest.
